# Longevity of outstanding sporting achievers: Mind versus muscle

**DOI:** 10.1371/journal.pone.0196938

**Published:** 2018-05-03

**Authors:** An Tran-Duy, David C. Smerdon, Philip M. Clarke

**Affiliations:** 1 Centre for Health Policy, School of Population and Global Health, University of Melbourne, Melbourne, Australia; 2 School of Economics, University of Queensland, Brisbane, Australia; Institute of Psychiatry, UNITED KINGDOM

## Abstract

**Background:**

While there is strong evidence showing the survival advantage of elite athletes, much less is known about those engaged in mind sports such as chess. This study aimed to examine the overall as well as regional survival of International Chess Grandmasters (GMs) with a reference to the general population, and compare relative survival (RS) of GMs with that of Olympic medallists (OMs).

**Methods:**

Information on 1,208 GMs and 15,157 OMs, respectively, from 28 countries were extracted from the publicly available data sources. The Kaplan-Meier method was used to estimate the survival rates of the GMs. A Cox proportional hazards model was used to adjust the survival for region, year at risk, age at risk and sex, and to estimate the life expectancy of the GMs. The RS rate was computed by matching each GM or OM by year at risk, age at risk and sex to the life table of the country the individual represented.

**Results:**

The survival rates of GMs at 30 and 60 years since GM title achievement were 87% and 15%, respectively. The life expectancy of GMs at the age of 30 years (which is near the average age when they attained a GM title) was 53.6 ([95% CI]: 47.7–58.5) years, which is significantly greater than the overall weighted mean life expectancy of 45.9 years for the general population. Compared to Eastern Europe, GMs in North America (HR [95% CI]: 0.51 [0.29–0.88]) and Western Europe (HR [95% CI]: 0.53 [0.34–0.83]) had a longer lifespan. The RS analysis showed that both GMs and OMs had a significant survival advantage over the general population, and there was no statistically significant difference in the RS of GMs (RS [95% CI]: 1.14 [1.08–1.20]) compared to OMs: (RS [95% CI]: 1.09 [1.07–1.11]) at 30 years.

**Conclusion:**

Elite chess players live longer than the general population and have a similar survival advantage to elite competitors in physical sports.

## Introduction

The writer Isaac Asimov once wrote that: “In life, unlike chess, the game continues after checkmate” [1]. In recent decades much research has been conducted into the longevity of a wide variety of sporting achievers. Almost all of the studies have been focused on a wide range of physical sports. A recent meta-analysis [2] and several recent reviews [3, 4] have consistently found that elite athletes engaged in physical sports (including soccer, baseball, cycling and various Olympic events) have a significant lower rate of mortality compared with the general population. The most comprehensive review, which involved nearly half a million individuals from 57 studies, indicates that the survival advantage for elite athletes was generally between 4 to 8 years longer [5].

Much less is known about those engaged in mind sports [6] such as chess where the mental exercise component dominates. A search for articles reporting longevity of players of mind sports in the Medline bibliographic database (see S1 Table for search strategy) identified only one early study involving 32 chess players born before 20th century [7]. This study [7] found that professional chess players had shorter lifespans than those players who had careers outside of chess and argued that this might be due to the mental strain of international chess competition. The study cited examples of three world champions who died prematurely from stroke [8]. More recently there are media reports of two competitors who died during a Chess Olympiad in Norway [9, 10]. When it comes to longevity, the news is not all bad for chess players, as a former professional player Zoltan Sarosy also held the title as Canada’s oldest living male [11]. However, such news stories bear little relationship to the average survival, as by definition news often involves unusual events such as premature mortality.

In the present study, we focused on survival of International Chess Grandmasters (GMs) which represent players, of whom most are professional, at the highest level. The Grandmaster title is based on rankings awarded to chess players by the World Chess Organization (FIDE) over many rounds of tournaments [12]. In its modern form the Grandmaster title was first awarded to 27 players in 1950 and there are now more than 1,500 players holding the Grandmaster title [13]. One advantage of studying elite chess players over competitors in other mind sports is that a system of rankings is maintained on an ongoing basis by FIDE [14], which is a way to track individuals over long periods, even after they retire from competition at the elite level.

Specific objectives of our study were to (1) examine the absolute overall as well as regional survival rates of GMs; (2) compare the life expectancy of GMs to the matched general population; and (3) compare the relative survival rates of GMs and Olympic medallists (OMs) who like many other physical sport competitors have been shown to have a survival advantage over the general population [15, 5].

## Materials and methods

### Data sources

#### Chess Grandmasters

Based on information from FIDE, Wikipedia publishes online information on GMs which, up to 1 Jan 2017, contains information on 1,711 players who achieved a Grandmaster title between 1950 and 2016 [13]. Variables in this dataset included dates of birth and death, year of Grandmaster title and affiliation country of the GMs [14]. Sex was not provided but based on another dataset of female GMs [16], we could identify the sex of all GMs.

#### Olympic medallists

Since the 1980s, the international consortium of historians and statisticians (OlyMADMen) has collected data on all Olympians. Earlier data were collected by one of the member of OlyMADMen and another co-worker in a research about Olympic Games [17]. Together, these data formed the OlyMADMen database, which contains information on 128,489 athletes who participated in Olympic Games between 1896 and 2016. Variables in this database included dates of birth, death and event, sex and countries represented, among others that are not relevant regarding our objectives. This information can now be accessed through the database on the International Olympic Committee website [18]. Further information on the data collection for the OlyMADMen database can be found in a recent study [15].

### Study cohorts

We considered a chess player with a Grandmaster title or an athlete that was awarded an Olympic medal as an outstanding achiever. To avoid immortal time bias [19], the date of this first achievement was defined as date at risk, i.e. the starting point of follow-up. In order to compare the relative survival of OMs with GMs, we restricted OMs to those that had been awarded medals in Games between 1950 and 2016. For OMs that achieved more than one medal, we retained only the record of the first medal.

From the Wikipedia and OlyMADMen databases, we extracted data on individuals from 30 countries of which available life tables covered the study period for at least 50 years. These life tables were obtained from the Human Mortality Database, an open online data source (see S2 Table for year coverage) [20]. Because only 7 GMs were from Oceanic region (6 from Australia and 1 from New Zealand), we excluded players from this region. As a result, the study cohorts of GMs and OMs contains data on 1,208 and 15,157 individuals, respectively, from 28 countries in three regions including North America (2 countries), Western Europe (16 countries) and Eastern Europe (10 countries). We found no match between any possible pair of GM and OM in terms of surname, given name, month of birth and year of birth, which indicated that there was no overlap between the two study cohorts. We also communicated with several chess historians to confirm this finding. The fact that no GM was also an OM is not surprising given the huge resources and dedication required to achieve membership of either of these elite groups.

### Statistical analysis

For each individual, the follow-up time was calculated as the duration between the date he or she became either a GM or an OM and date of death or last date of data collection (i.e. 1 Jan 2017), whichever came first. As longevity might be influenced by geographic areas and years at risk, we created dummy variables indicating regions (North America, Western Europe or Eastern Europe) and periods of achieving the Grandmaster title (1950–1970, 1970–1990 or 1990–2017). Then, we used the Kaplan-Meier estimator to examine the overall survival of GMs as well as survival of GMs in each region and each period, and the Cox proportional hazards (PHs) model to adjust the survival for region, year at risk, age at risk and sex. For the latter, the PHs assumption was tested using the scaled Schoenfeld residuals [21]. The fitted Cox PHs model was used to construct adjusted survival curves for male GMs with a fixed year at risk of 2010 and five-year age groups in different regions. We calculated the adjusted life expectancy of these GMs as the areas under the survival curves using a truncated estimator [22], which were compared with that in the life tables matched by year, age and region. We chose 2010 as the year at risk for this computation because this is the most recent year in which the life tables were available for all countries. We used the bootstrap method to compute the 95% confidence intervals of the GMs’ life expectancy, where data were sampled (with replacement) for 10,000 times and to each bootstrapped data set a Cox PH model was fitted and used for computing the life expectancy as described above. The weighted mean life expectancy of the general population for each region was calculated using the population of each member country as a weight.

To compare the survival rates between GMs, OMs and the general population, we computed the “relative survival rate”, which was the ratio of the observed survival rate to the expected survival rate, at different time points over the follow-up period. The observed survival rates were estimated using the Kaplan-Meier estimator, and the expected survival rates by matching each GM or OM by year at risk, age at risk and sex to the life table of the country the individual represented, based on which a survival curve was constructed using the method described by Hakulinen [23]. For example, if a Canadian woman achieved the Grandmaster title in 2000 at the age of 25, the follow-up time of this person and the national mortality rates from age 25 onward in the life table of the Canadian female population in 2000 would be used to compute the expected survival. We used the function survexp in the R package survival [24] for this purpose, which estimates, for each individual, the total hazard experienced up to its observed death or last follow-up time, and combines the individual expected survival curves to produce an overall survival curve [23]. We used the conditional survival technique to adjust the expected survival rate so that it is unaffected by unidentified loss to follow-up [25]. The 95% confidence intervals of the relative survival rates were calculated based on Fieller’s theorem [26].

## Results

Table 1 provides information on characteristics of GMs and OMs in 28 countries and three regions. Most of the GMs (59%) were from Eastern Europe, and only 9% were from North America. In contrast, Western Europe accounted for the largest proportion of OMs (47%), which was followed by Eastern Europe (30%). Mean ages at Grandmaster title were similar among regions (range: 27.2–29.0). Interestingly, these values were relatively close to the mean ages at first Olympic medal (range: 25.4–26.2). Up to 97–99% of the GMs were male, while these percentages in OMs were much lower, ranging from 57% to 69% depending on regions. By 1 Jan 2017, the numbers of deaths (% within region) in North America, Western Europe and Eastern Europe were 16 (14.3%), 28 (7.3%) and 79 (11.1%) among GMs, respectively, and 400 (8.2%), 1049 (10.1%) and 1256 (19.0%) among OMs, respectively. Mean follow-up times (SDs) of GMs in North America, Western Europe and Eastern Europe were 22.3 (14.8), 18.3 (12.2) and 16.6 (11.6), respectively. Mean follow-up times (SDs) of OMs were longer than of GMs, which were 23.9 (17.6), 25.4 (17.1) and 16.6 (11.6) in North America, Western Europe and Eastern Europe, respectively. On average, a chess player achieved the Grandmaster title (mean year: 1997.3) about a decade later than an OM obtaining the first medal (mean year: 1988.4).

**Table 1:** Characteristics of chess Grandmasters and Olympic medallists (1950–2016).

	Number of individuals (% within the same grouping level)		Age at first achievement^a^, mean (SD)		Year at first achievement^a^, range (mean)		Follow-up time, mean (SD)		Male, n (%)	
	GM	OM	GM	OM	GM	OM	GM	OM	GM	OM
**North America**	**112 (9)**	**3368 (22)**	**27.6 (12.1)**	**24.9 (5.1)**	**1950–2016 (1992.1)**	**1952–2016 (1989.4)**	**22.3 (14.8)**	**25.6 (17.8)**	**110 (98)**	**2054 (61)**
Canada	14 (12)	772 (23)	26.1 (11.1)	25.8 (5.6)	1964–2016 (1998.4)	1952–2016 (1993.6)	16.3 (13.5)	22.0 (17.0)	14 (100)	435 (56)
USA	98 (88)	2596 (77)	27.8 (12.3)	24.6 (4.9)	1950–2015 (1991.2)	1952–2016 (1988.2)	23.1 (14.9)	26.6 (1798)	96 (98)	1619 (62)
**Western Europe**	**386 (32)**	**7194 (47)**	**29.0 (10.6)**	**25.9 (5.0)**	**1950–2016 (1997.0)**	**1952–2016 (1988.3)**	**18.3 (12.2)**	**26.4 (17.4)**	**382 (99)**	**5043 (70)**
Austria	9 (2)	199 (3)	30.9 (8.0)	25.7 (4.7)	1952–2008 (1988.7)	1952–2016 (1986.5)	23.9 (12.5)	29.2 (18.1)	9 (100)	147 (74)
Belgium	9 (2)	80 (1)	32.5 (12.4)	25.3 (4.3)	1956–2015 (1994.8)	1952–2016 (1992.0)	17.2 (9.5)	22.2 (20.4)	9 (100)	59 (74)
Denmark	13 (4)	227 (3)	28.8 (11.7)	27.0 (5.2)	1956–2016 (1998.7)	1952–2016 (1991.1)	17.4 (14.5)	23.6 (18.3)	13 (100)	154 (68)
Finland	6 (2)	360 (5)	34.4 (20.4)	26.4 (5.1)	1975–2003 (1989.0)	1952–2016 (1985.0)	23.1 (13.9)	28.2 (18.0)	6 (100)	282 (78)
France	50 (13)	682 (9)	27.0 (8.3)	26.4 (5.0)	1978–2016 (2001.5)	1952–2016 (1992.3)	15.1 (9.2)	22.5 (17.7)	49 (98)	519 (76)
Germany	95 (25)	2095 (29)	29.9 (10.1)	24.7 (4.6)	1952–2016 (1995.6)	1952–2016 (1985.0)	19.8 (14.1)	30.4 (15.6)	95 (100)	1379 (66)
Iceland	13 (3)	17 (0)	25.5 (4.3)	27.5 (3.6)	1958–2013 (1991.2)	1956–2008 (2003.1)	25.4 (14.9)	13.3 (13.5)	13 (100)	16 (94)
Ireland	1 (0)	26 (0)	29.3 (NA)	24.8 (4.2)	1996–1996 (1996.0)	1952–2016 (1989.7)	20.6 (NA)	25.2 (22.3)	1 (100)	22 (85)
Italy	14 (4)	743 (10)	35.2 (22.4)	26.0 (4.7)	1974–2015 (2000.2)	1952–2016 (1986.8)	14.1 (12.1)	27.0 (18.4)	14 (100)	621 (84)
Netherlands	37 (10)	490 (7)	26.5 (10.7)	25.6 (4.3)	1950–2015 (1999.1)	1952–2016 (1993.6)	15.4 (11.1)	21.9 (15.7)	36 (97)	249 (51)
Norway	13 (3)	339 (5)	27.6 (11.4)	26.2 (4.5)	1985–2016 (2003.4)	1952–2016 (1990.0)	13.2 (8.9)	25.2 (16.3)	13 (100)	203 (60)
Portugal	3 (1)	20 (0)	36.1 (3.7)	27.7 (5.5)	1994–2002 (1999.3)	1952–2016 (1990.0)	17.3 (4.6)	25.8 (20.2)	3 (100)	16 (80)
Spain	47 (12)	376 (5)	28.4 (7.1)	25.9 (4.7)	1962–2016 (1999.4)	1952–2016 (1998.0)	16.7 (12.1)	18.0 (12.5)	47 (100)	265 (70)
Sweden	24 (6)	567 (8)	31.7 (12.6)	27.3 (5.8)	1950–2016 (1994.1)	1952–2016 (1984.2)	17.1 (10.9)	28.9 (18.4)	23 (96)	436 (77)
Switzerland	8 (2)	290 (4)	25.1 (5.0)	28.3 (6.7)	1956–2009 (1990.5)	1952–2016 (1987.0)	24.9 (15.7)	26.2 (18.0)	8 (100)	212 (73)
UK	44 (11)	683(9)	28.3 (10.2	26.4 (5.4)	1976–2014 (1994.3)	1952–2016 (1990.7)	21.5 (9.4)	24.3 (19.0)	43 (98)	463 (68)
**Eastern Europe**	**710 (59)**	**4595 (30)**	**27.2 (9.4)**	**25.2 (4.2)**	**1950–2016 (1998.3)**	**1952–2016 (1984.4)**	**16.6 (11.6)**	**29.0 (16.8)**	**691 (97)**	**3242 (71)**
Bulgaria	38 (5)	280 (6)	28.7 (5.9)	24.6 (3.9)	1961–2013 (1993.9)	1952–2016 (1980.9)	21.5 (13.4)	33.5 (12.8)	37 (97)	184 (66)
Czech Republic	33 (5)	406 (9)	29.0 (8.9)	25.7 (3.9)	1955–2014 (1998.2)	1952–2016 (1980.3)	17.8 (12.5)	33.7 (15.9)	33 (100)	335 (83)
Estonia	7 (1)	17 (0)	27.4 (10.6)	29.9 (3.5)	1990–2016 (2003.7)	1992–2016 (2004.1)	10.4 (6.3)	12.3 (8.0)	7 (100)	15 (88)
Hungary	61 (9)	537 (12)	27.1 (10.2)	25.5 (4.7)	1950–2016 (1993.3)	1952–2016 (1975.5)	21.0 (15.8)	36.0 (16.3)	59 (97)	411 (77)
Latvia	15 (2)	23 (1)	36.3 (17.9)	27.0 (4.7)	1957–2012 (1993.9)	1992–2014 (2006.0)	18.3 (9.5)	10.6 (7.1)	15 (100)	21 (91)
Lithuania	9 (1)	50 (1)	34.9 (16.8)	26.9 (4.4)	1984–2013 (1995.8)	1992–2016 (2001.5)	18.1 (10.2)	14.8 (8.5)	8 (89)	40 (80)
Poland	41 (6)	379 (8)	24.3 (7.8)	25.5 (3.8)	1976–2016 (2003.1)	1952–2016 (1981.3)	13.2 (8.5)	32.5 (15.6)	40 (98)	300 (79)
Russia	407 (57)	2714 (59)	27.1 (9.5)	25.0 (4.1)	1950–2016 (1998.2)	1952–2016 (1985.6)	16.2 (11.1)	27.7 (16.8)	397 (98)	1833 (68)
Ukraine	89 (13)	164 (4)	25.4 (6.6)	25.8 (4.3)	1967–2015 (2002.0)	1994–2016 (2004.4)	14.2 (9.3)	11.8 (6.2)	85 (96)	83 (51)
Slovakia	10 (1)	25 (1)	28.9 (4.4)	26.2 (4.6)	1978–2016 (1997.7)	1996–2016 (2006.5)	18.9 (13.9)	9.9 (6.9)	10 (100)	20 (80)
**Overall**	**1208 (100)**	**15157 (100)**	**27.8 (10.1)**	**25.5 (4.8)**	**1950–2016 (1997.3)**	**1952–2016 (1987.4)**	**17.7 (12.2)**	**27.0 (17.4)**	**1183 (98)**	**10339 (68)**

Abbreviations: GM, chess Grandmaster; OM, Olympic medallist

^a^ First achievement means Grandmaster title for chess players or first medal for Olympic medallists

**Fig 1:**
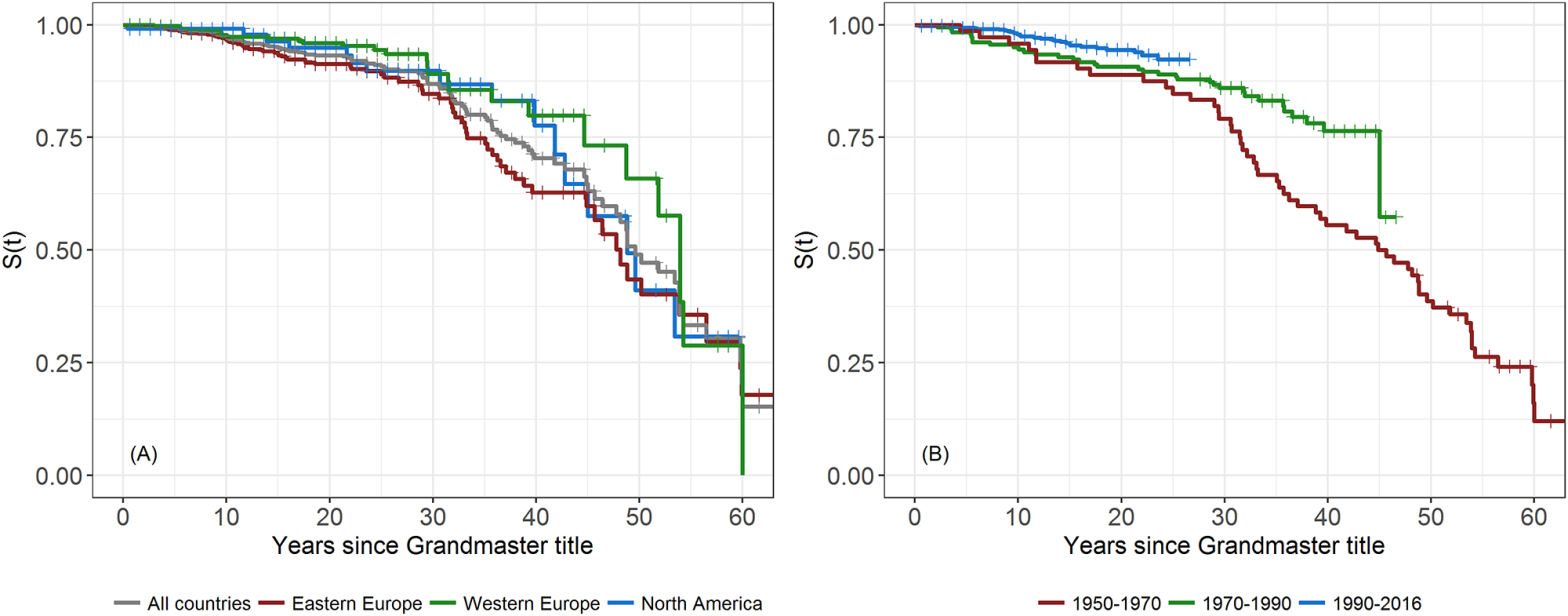
Kaplan-Meier plots of survival of GMs in (A) all countries and different regions, and (B) periods of achieving the Grandmaster title.

Fig 1A shows the Kaplan-Meier (K-M) plots of overall and regional survival of GMs, and Fig 1B shows the K-M survival curves of GMs in different periods of achieving the Grandmaster title. The overall survival rates of GMs at 10, 30 and 60 years since achievement of the GM title were 97%, 87% and 15%, respectively. The GMs in Western Europe and North America appeared to have a survival advantage over those in Eastern Europe. Survival rates of the GMs in the period 1990–2016 [mean age (SD): 26.7 (9.1)] were greater than that of the GMs in the period 1970–1990 [mean age (SD): 31.6 (13.9)], whose survival rates were greater than that of the GMs in the period 1950–1970 [mean age (SD): 32.7 (7.7)].

In fitting the Cox PH model, the beta coefficient for sex was not significant. Exclusion of sex changed the beta coefficients of other variables by less than 2% and did not change their statistical significance. Therefore, we included only region, year at risk and age at risk in the final model. The PHs assumption was satisfied by the scaled Schoenfeld residuals test. The results from the Cox PHs regression indicated that compared to Eastern Europe, GMs in North America (adjusted hazard ratio [95% CI]: 0.51 [0.29–0.88]) and Western Europe (adjusted hazard ratio [95% CI]: 0.53 [0.34–0.83]) had a longer lifespan, which confirmed the interpretation of the Kaplan-Meier plot (Fig 1A). Survival of GMs in North America was not significantly different from that in Western Europe (adjusted hazard ratio [95% CI]: 0.95 [0.51–1.77]). Detailed outputs from the Cox regression are provided in S3 Table.

Table 2 presents overall and regional life expectancy of a male GM who obtained the Grandmaster title in 2010 at an age in the range of 25–100 years, and life expectancy of the general male population matched by age and region. The overall life expectancy of GMs at the age of 30 years (which is near the average age when they attained a GM title) was 53.6 ([95% CI]: 47.7–58.5) years, which is significantly greater than the overall weighted mean life expectancy of 45.9 years for the general population. In all three regions, mean life expectancy of the GMs was longer than that of the matched general population, with gaps between them ranging from 1 to 14 years depending on age.

**Table 2:** Life expectancy in 2010 of male chess Grandmasters (GMs) in different regions compared with the observed life expectancy of the general male populations among countries in the corresponding regions.

Age	All countries		Eastern Europe		Western Europe		North America	
	GMs, mean (95% CI)	General population, weighted mean (min-max)	GMs, mean (95% CI)	General population, weighted mean (min-max)	GMs, mean (95% CI)	General population, weighted mean (min-max)	GMs, mean (95% CI)	General population, weighted mean (min-max)
25	56.8 (51.8–60.7)	50.5 (39.8^a^—55.7^b^)	56.2 (49.8–60.0)	42.4 (39.8^a^—50.1^g^)	59.8 (55.0–62.2)	54.0 (52.4^i^—55.7^b^)	60.0 (54.1–62.7)	52.8 (52.6^o^—55.1^c^)
30	53.6 (47.7–58.5)	45.9 (35.6^a^, 50.8^b^)	52.3 (45.1–57.3)	38.1 (35.6^a^ —45.34^g^)	57.1 (51.4–60.7)	49.2 (47.6^i^—50.8^b^)	57.5 (50.0–61.5)	48.1 (47.9^o^—50.3^c^)
35	49.5 (42.9–55.3)	41.3 31.8^a^, 45.9^b^)	47.3 (39.9–53.5)	33.9 (31.8^a^ —40.5^g^)	53.5 (46.8–58.4)	44.4 (42.8^i^—45.9^b^)	54.0 (45.2–59.5)	43.5 (43.2^o^—45.5^c^)
40	44.6 (37.9–51.1)	36.7 (27.9^a^, 41.1^b^)	41.6 (34.6–48.5)	29.8 (27.9^a^ —35.8^g^)	48.9 (41.5–55.0)	39.6 (38.1^i^—41.1^b^)	49.4 (40.0–56.7)	38.8 (38.6^o^—40.7^c^)
45	39.2 (32.9–46.0)	32.3 (24.2^a^, 36.4^b^)	35.8 (29.4–42.6)	25.8 (24.4^a^ —31.2^g^)	43.4 (36.2–50.5)	34.9 (33.6^k^—36.4^b^)	44.0 (34.6–52.8)	34.2 (34.1^o^—36.0^c^)
50	33.8 (28.0–40.3)	28.0 (20.7^a^, 31.7^b^)	30.2 (24.6–36.4)	22.1 (20.7^a^ —26.7^g^)	37.5 (31.0–45.0)	30.4 (29.1^k^—31.7^b^)	38.2 (29.5–47.7)	29.9 (29.7^o^—31.4^c^)
55	28.5 (23.4–34.5)	24.0 (17.5^a^, 27.2^b^)	24.9 (20.1–30.2)	18.7 (17.5^a^ —22.6^g^)	31.8 (26.0–39.0)	26.0 (24.8^k^—27.2^b^)	32.4 (24.7–41.7)	25.7 (25.3^o^—27.0^c^)
60	23.6 (19.1–28.8)	20.1 (14.6^a^, 22.9^b^)	20.1 (15.9–24.6)	15.6 (14.6^a^ —18.8^g^)	26.4 (21.3–32.9)	21.9 (20.8^k^—22.9^b^)	27.0 (20.1–35.5)	21.7 (21.6^o^—22.8^c^)
65	19.0 (15.1–23.6)	16.6 (12.0^a^, 18.8^b^)	15.9 (12.3–19.6)	12.8 (12.0^a^ —15.3^g^)	21.5 (17.1–27.1)	17.9 (16.9^k^—18.8^b^)	22.0 (15.9–29.4)	17.9 (17.8^o^—18.7^c^)
70	15.0 (11.6–18.9)	13.3 (9.6^a^, 15.0^c^)	12.2 (9.2–15.3)	10.2 (9.6^a^ —12.2^g^)	17.1 (13.3–21.8)	14.3 (13.3^k^—14.9^m^)	17.5 (12.2–23.8)	14.4 (14.3^o^—15.0^c^)
75	11.6 (8.7–14.9)	10.2 (7.5^d^, 11.5^c^)	9.3 (6.8–11.8)	8.0 (7.5^d^—9.4^h^)	13.2 (10.0–17.2)	10.9 (10.2^k^—11.5^m^)	13.6 (9.1–18.8)	11.2 (11.1^o^—11.5^c^)
80	8.9 (6.4–11.6)	7.6 (5.7^d^, 8.5^c^),	7.1 (4.9–9.1)	6.1 (5.7^d^ —7.1^f^)	10.1 (7.4–13.3)	8.0 (7.4^i^—8.5^m^)	10.4 (6.8–14.6)	8.3 (8.3^o^—8.5^c^)
85	6.8 (4.6–9.1)	5.4 (4.2^e^, 6.0^c^)	5.4 (3.4–7.2)	4.5 (4.2^e^—5.3^f^)	7.7 (5.4–10.2)	5.6 (5.2^l^—5.9^m^)	7.9 (4.9–11.1)	5.9 (5.9^o^—6.0^c^)
90	5.2 (3.2–7.2)	3.8 (2.8^e^, 4.2^f^)	4.0 (2.2–5.8)	3.3 (2.8^e^ —4.2^f^)	5.8 (3.8–8.0)	3.9 (3.4^l^—4.1^m^)	6.0 (3.4–8.6)	4.1 (4.0^o^—4.2^c^)
95	4.0 (2.1–5.9)	2.7 (2.1^e^, 3.2^f^)	2.9 (1.3–4.7)	2.5 (2.1^e^ —3.2^f^)	4.4 (2.5–6.4)	2.7 (2.3^l^—2.8^n^)	4.5 (2.2–6.8)	2.8 (2.8^o^—2.9^c^)
100	3.0 (1.2–4.9)	2.0 (1.6^e^, 2.6^f^)	2.1 (0.6–4.0)	1.2 (1.6^e^ —2.6^f^)	3.2 (1.5–5.2)	1.9 (1.7^l^—2.1^n^)	3.3 (1.3–5.5)	2.1 (2.0^o^—2.1^c^)

Abbreviation: GMs, chess Grandmasters; CI, confidence interval

^a^ Russia;

^b^ Switzerland;

^c^ Canada;

^d^ Ukraine;

^e^ Bulgaria;

^f^ Latvia;

^g^ Czech Republic;

^h^ Poland;

^i^ Portugal;

^k^ Denmark;

^l^ Iceland;

^m^ France;

^n^ Spain;

^o^ USA

**Fig 2:**
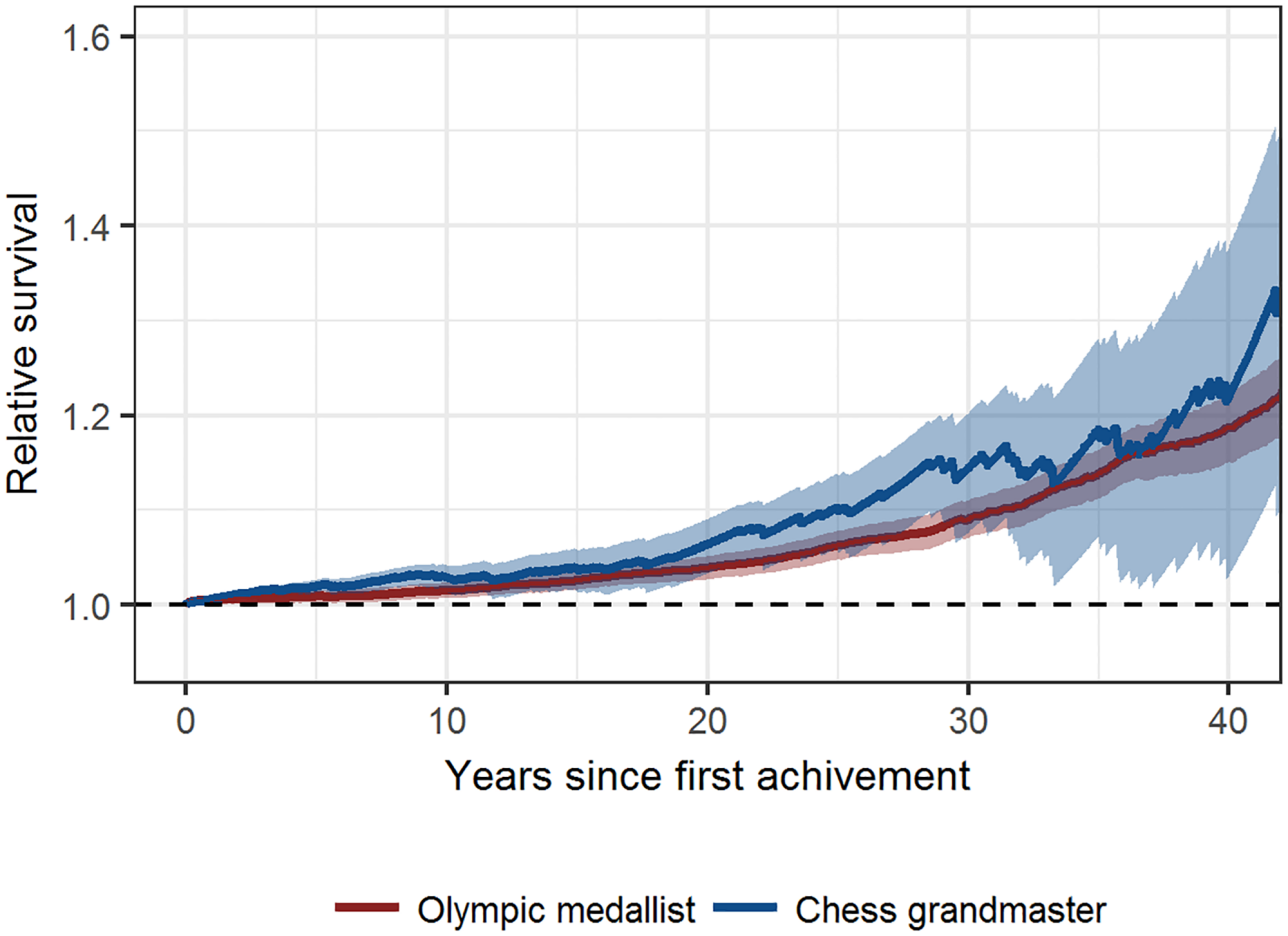
Survival of chess Grandmasters and Olympic medallists from 28 countries relative to the general population from the same countries. The lines represent the ratios of the observed survival rates to the expected survival rates. The shaded areas represent the 95% confidence regions. First achievement means the achievement of the Grandmaster title for chess players or of the first medal for Olympic athletes.

Fig 2 shows the survival of GMs and OMs from 28 countries relative to the general population from the same countries. Both GMs and OMs had a significant survival advantage over the general population. There was no statistically significant difference in the relative survival of GMs vs OMs. At 10, 20 and 30 years, the relative survival rates [95% CI] of GMs were 1.03 [1.01–1.04], 1.06 [1.03–1.09] and 1.14 [1.08–1.20], respectively, and of OMs were 1.02 [1.01–1.03], 1.04 [1.03–1.05] and 1.09 [1.07–1.11], respectively. Stratified regional analyses (S1 Fig) did not indicate significant differences from the overall analyses.

## Discussion

While the sports science literature has often featured the link between longevity and elite competitors in physical sports [5], we found a similar association in a *mind* sport. The elite mental athletes in our study, GMs, had a substantially higher life expectancy than the general population in each of the three regions studied. Across the combined sample from 28 countries, the survival advantage over the general population significantly increased over time. To the best of our knowledge, the present study is the first that compared the longevity of chess players with that of the general population and athletes using advanced statistical methods.

Our results are contradictory to the early study identified in our review of the previous literature [7], which may be attributed to the differences in sample size and analytical techniques. We used a sample of 1,208 players, while the earlier study included only 32 players. The positive effects of chess sport on longevity are particularly interesting in an era in which the so-called ‘mind sports’ (chess, Go, Shogi), poker and eSports (competitive video games) have become highly professionalized. Given that competitive chess is a stressful activity that creates measurable physiological tension [27], and following the anecdotal evidence of its negative health effects reported in the introduction, it is worth identifying the sources of longevity benefits that outweigh these potential harms. Additionally, one question that our study cannot answer is whether proficiency in cognitive sports causes an increased life expectancy, or whether one or more confounding factors are driving the effect. While our study was not designed to answer this, there are some potential mechanisms that may play a role in explaining the association between playing chess and longevity.

While intelligence may be a potential confounding factor given its positive effect on longevity [28, 29], evidence of the link between IQ and chess ability is inconclusive. Several studies have failed to find a superiority of expert chess players in a variety of intellectual dimensions, including selective attention, inhibition and executive cognitive function [30], logical and computational skills [31], visual memory [32], and complex planning [33]. Although other studies have found a positive relationship between intelligence and chess skill [34, 35], they also showed that this effect disappeared or even reversed at higher levels of chess expertise [36]. Therefore, the impact of the absence of adjustment for IQ on our study outcomes would be small.

A more likely channel is that to attain the Grandmaster title an individual may be encouraged to make necessary health improvements (e.g. reduced smoking and alcohol consumption, improved nutrition, more regular cardiovascular exercise, etc.) to improve one’s cognitive performance. Although there has been some concern that chess training promotes a sedentary lifestyle that may reduce participation of the chess players in physical activities, this is not supported by existing evidence. The importance of physical exercise and healthy diet for professional chess is well known amongst GMs, and world championship contenders normally employ a full-time nutritionist and/or physical trainer in preparation for and during world championship matches [37]. While the frequency of health and fitness activities conducted by chess players is apparently less than that by athletes excelling in Olympic sports, there is evidence suggesting that chess players do exhibit a higher level of physical fitness than the general population [38]. The longevity of Olympians has been attributed to factors such as long-term vigorous exercise training [3] and thus it is notable that chess players who do not attain this level of physical training have a similar survival advantage over the general population.

Additionally, there may be potential direct health benefits of chess expertise. There is evidence suggesting that playing chess can reduce the risk of dementia [39], as well as physically alter the structure of the brain [40–42]. It is also possible that attaining the exalted Grandmaster title may in itself increase life expectancy through psychological payoffs, which follows a body of literature on the connection between longevity and “outstanding achievement” [43]. Hence, when it comes to predicting longevity both fitness of mind and muscle appear to be important.

Another causal argument on the effect of developing chess expertise on survival relates to socioeconomic mechanisms. Becoming a chess grandmaster may provide an economic and social boost, which has been strongly linked to increased life expectancy [44, 45]. The relative income and social status benefits of GMs are plausibly highest for individuals in Eastern Europe, which would explain the particularly substantial relative survival advantage we found in this region. In the Soviet Union, for example, becoming a professional chess player was supported by the State and playing chess at a master level promoted as an esteemed profession [46]. Today, degrees in higher education with chess specializations are available in Russia, largely through State-funded scholarships.

Our study has some limitations. The data were obtained from online databases which involve ascertaining deaths largely through passive follow-up (e.g. media reports). A recent study on mortality of French Olympians has used national registry data to ascertain the death dates of the participants which may provide a means of reducing the loss to follow-up [47]. Given that our study involves 28 countries, it would be hard to replicate this approach in this study. We did examine the number of missing death dates for OMs born prior to 1917, i.e. those who are centenarians if they are alive. We found only four of the 115 OMs with missing deaths and for only one of these four OMs we found information indicating that he had died, i.e. information on death that was not recorded in the OlyMADMen database. This finding suggests that the fraction of OMs who were lost to follow-up is most likely small and this should be accounted for using the conditional relative survival methods which have been used in a previous analysis of OMs [15]. Another limitation is that the data sources used for the analyses do not contain variables such as causes of death, education level, socioeconomic status, levels of physical exercises and health behaviours that may help to explain survival advantage of the GMs. Moreover, in observational studies it cannot be excluded that there are unknown factors that confound the results. We focused on GMs as elite chess players, i.e. those who attained a FIDE rating of 2500 or higher. Although this cut-off point is meaningful for the recognition of the Grandmaster title, it does not necessarily signify a threshold of performance which differentiates the life expectancy. To have a more in-depth picture of the link between chess performance and longevity would require the analysis of a larger sample of chess players with different levels of achievement. Future directions of research could also focus on exploring the different mechanisms that may link chess expertise and longevity, particularly the direct physical effects on brain structure and the role of socioeconomic status.

## Conclusion

Not only does the game of life continue after the checkmate, but excelling in mind sports like chess means one is likely to play the game for longer.

## Supporting information

S1 Fig Survival of chess Grandmasters and Olympic medallists relative to the general population in different regions. (PDF)

S1 Table Search strategy for studies reporting longevity of players of mind sports. (DOCX)

S2 Table Life table coverage. (DOCX)

S3 Table Output from Cox proportional hazard regression on survival time of chess Grandmasters. (DOCX)
