# Supplementary material for: Longevity of outstanding sporting achievers: Mind versus muscle
Source: PLoS One. 2018 May 3;13(5):e0196938. doi: 10.1371/journal.pone.0196938 (PMC5933783; doi:10.1371/journal.pone.0196938)
Supplement: S1 Fig — (PDF) [file pone.0196938.s001.pdf]

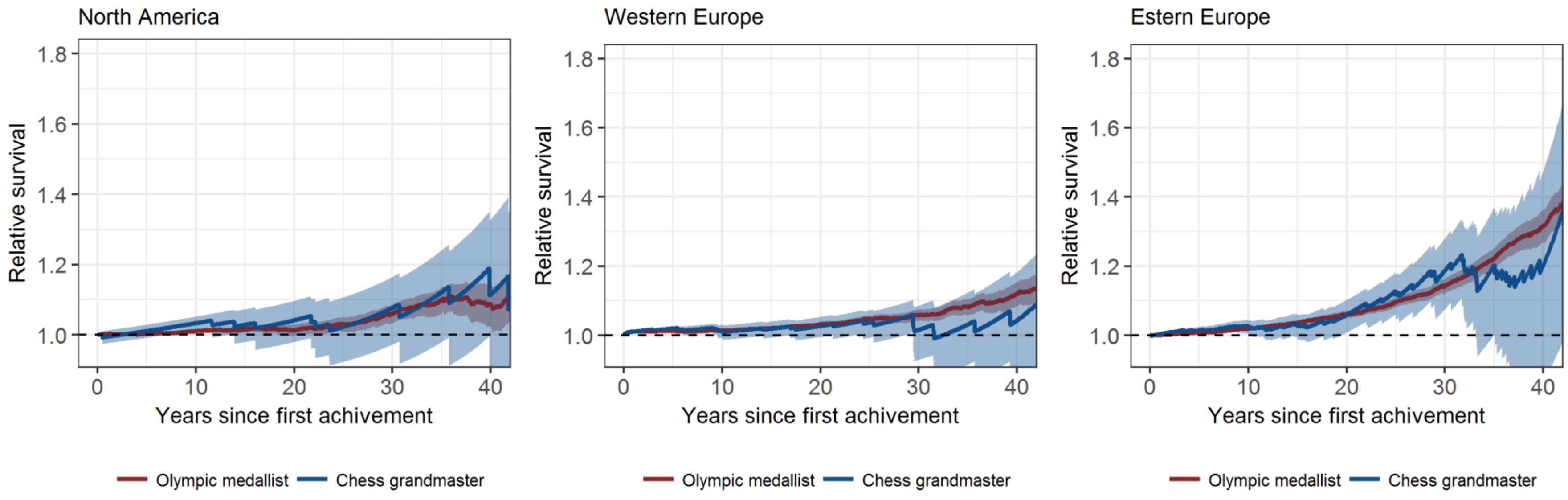


**S1 Fig. Survival of chess Grandmasters and Olympic medalists relative to the general population in different regions.** The lines represent the ratios of the observed survival rates to the expected survival rates. The shaded areas represent the 95% confidence regions. First achievement means the achievement of the Grandmaster title for chess players or of the first medal for Olympic athletes.
