# Supplementary material for: Longevity of outstanding sporting achievers: Mind versus muscle
Source: PLoS One. 2018 May 3;13(5):e0196938. doi: 10.1371/journal.pone.0196938 (PMC5933783; doi:10.1371/journal.pone.0196938)
Supplement: S1 Table — (DOCX) [file pone.0196938.s002.docx]

**S1 Table. Search strategy for studies reporting longevity of players of mind sports.** Repeated searches were conducted on 20 March 2017 using the terms “survival”, or “life expectancy”, or “longevity”, or “mortality” combined with six popular mind sports: Chess, Draughts, Bridge, Poker, Backgammon and Go. For the games Bridge and Go, the word “game” was added as an additional qualifier to delineate these terms from medical uses. Abstracts were scanned for relevance. The search identified 13 studies, of which only one article was relevant.

| **Search terms** | **Results** |
| --- | --- |
| (survival[Title/Abstract]) AND chess[Title/Abstract]) | 6 articles, none relevant |
| ("life expectancy"[Title/Abstract]) AND chess[Title/Abstract] | 0 articles |
| ("longevity"[Title/Abstract]) AND chess[Title/Abstract] | 1 article, **1 relevant** |
| ("survival"[Title/Abstract]) AND draughts[Title/Abstract] | 0 article |
| ("life expectancy"[Title/Abstract]) AND draughts[Title/Abstract] | 0 article |
| ("longevity"[Title/Abstract]) AND draughts[Title/Abstract] | 0 article |
| ("mortality"[Title/Abstract]) AND draughts[Title/Abstract] | 0 article |
| ("survival"[Title/Abstract]) AND game[Title/Abstract] AND bridge[Title/Abstract] 2 articles, | 0 relevant |
| ("life expectancy"[Title/Abstract]) AND game[Title/Abstract] AND bridge[Title/Abstract] | 0 articles |
| ("longevity"[Title/Abstract]) AND game[Title/Abstract] AND bridge[Title/Abstract] | 1 article, 0 relevant |
| ("mortality"[Title/Abstract]) AND game[Title/Abstract] AND bridge[Title/Abstract] | 1 article, 0 relevant |
| ("survival"[Title/Abstract]) AND poker[Title/Abstract] | 2 article, 0 relevant |
| ("longevity"[Title/Abstract]) AND poker[Title/Abstract] | 0 article |
| ("life expectancy"[Title/Abstract]) AND poker[Title/Abstract] | 0 article |
| ("mortality"[Title/Abstract]) AND poker[Title/Abstract] | 0 article |
| ("survival"[Title/Abstract]) AND backgammon[Title/Abstract] | 0 article |
| ("life expectancy"[Title/Abstract]) AND backgammon[Title/Abstract] | 0 article |
| ("longevity"[Title/Abstract]) AND backgammon[Title/Abstract] | 0 article |
| ("mortality"[Title/Abstract]) AND backgammon[Title/Abstract] | 0 article |
| ("survival"[Title/Abstract]) AND Go [Title/Abstract] AND game [Title/Abstract] | 0 article |
| ("life expectancy"[Title/Abstract]) AND Go [Title/Abstract] AND game [Title/Abstract] | 0 article |
| ("longevity"[Title/Abstract]) AND Go [Title/Abstract] AND game [Title/Abstract] | 0 article |
| ("mortality"[Title/Abstract]) AND Go [Title/Abstract] AND game [Title/Abstract] | 0 article |
