# Supplementary material for: Longevity of outstanding sporting achievers: Mind versus muscle
Source: PLoS One. 2018 May 3;13(5):e0196938. doi: 10.1371/journal.pone.0196938 (PMC5933783; doi:10.1371/journal.pone.0196938)
Supplement: S2 Table — (DOCX) [file pone.0196938.s003.docx]

**S2 Table. Life table coverage.**

| Country | Year coverage^a^ | Unavailable |
| --- | --- | --- |
| *North America* |  |  |
| Canada | 1921 – 2011 (1 x 1) |  |
| USA | 1933 – 2014 (1 x 1) |  |
| *Western European* |  |  |
| Austria | 1947 – 2014 (1 x 1) |  |
| Belgium | 1919 – 2015 (1 x 1) |  |
| Denmark | 1835 – 2014 (1 x 1) |  |
| Finland | 1878 – 2015 (1 x 1) |  |
| France | 1816 – 2014 (1 x 1) |  |
| Germany | 1956 – 2013 (1 x 1) | 1950 – 1955 |
| Iceland | 1838 – 2013 (1 x 1) |  |
| Ireland | 1950 – 2014 (1 x 1) |  |
| Italy | 1872 – 2012 (1 x 1) |  |
| Netherlands | 1850 – 2012 (1 x 1) |  |
| Norway | 1846 – 2014 (1 x 1) |  |
| Portugal | 1940 – 2012 (1 x 1) |  |
| Spain | 1908 – 2014 (1 x 1) |  |
| Sweden | 1751 – 2014 (1 x 1) |  |
| Switzerland | 1876 – 2014 (1 x 1) |  |
| UK | 1922 – 2013 (1 x 1) |  |
| *Eastern European* |  |  |
| Bulgaria | 1947 – 2010 (1 x 1) |  |
| Czech Republic | 1950 – 2014 (1 x 1) |  |
| Estonia | 1959 – 2013 (1 x 1) | 1950 – 1958 |
| Hungary | 1950 – 2014 (1 x 1) |  |
| Latvia | 1959 - 2013 (1 x 1) | 1950 – 1958 |
| Lithuania | 1959 - 2013 (1 x 1) | 1950 – 1958 |
| Poland | 1952 (1 x 1), 1958 – 2014 (1 x 1) | 1953 – 1957 |
| Russia | 1956-2014 (1 x 1) | 1950 – 1955 |
| Slovakia | 1950 – 2014 (1 x 1) |  |
| Ukraine | 1959 – 2013 (1 x 1) | 1950 – 1958 |

Source: [www.mortality.org](http://www.mortality.org)

^a^ (n x m): n is the age interval within each life table, m is the calendar year interval between life tables
