# Supplementary material for: Longevity of outstanding sporting achievers: Mind versus muscle
Source: PLoS One. 2018 May 3;13(5):e0196938. doi: 10.1371/journal.pone.0196938 (PMC5933783; doi:10.1371/journal.pone.0196938)
Supplement: S3 Table — (DOCX) [file pone.0196938.s004.docx]

**S3 Table. Output from Cox proportional hazard regression on survival time of chess Grandmasters.**

| Covariate | Coef | Hazard ratio (HR) | SE (coef) | 95% CI (HR) | P-value (coef) |
| --- | --- | --- | --- | --- | --- |
| Western Europe | 0.62900 | 0.5331 | 0.2248 | 0.3431-0.8284 | 0.00515 |
| North America | -0.6812 | 0.5060 | 0.2835 | 0.2903-0.8821 | 0.01628 |
| Year at GM title | -0.0155 | 0.9846 | 0.0074 | 0.9705-0.9990 | 0.03641 |
| Age at GM title | 0.0003 | 1.0003 | 0.00002 | 1.0002-1.0003 | < 0.0001 |

Concordance = 0.803 (se = 0.035); R^2^ = 0.148 (max possible = 0.667); Likelihood ratio test = 193.1 on 4 df, p = 0; Wald test = 233.7 on 4 df, p = 0; Score (logrank) test = 350.6 on 4 df, p = 0
